# Soft tissue abnormalities in the congenital limb malformation radial dysplasia (RD): Their clinical impact and treatment significance

**DOI:** 10.1111/joa.70118

**Published:** 2026-02-13

**Authors:** Marco Correia Duarte, Sultan M. Al‐Zyoud, Eleanor M. Feneck, Duane James, Gillian D. Smith, Branavan Sivakumar, Malcolm P. O. Logan

**Affiliations:** ^1^ Randall Centre for Cell and Molecular Biophysics King's College London London UK; ^2^ UCL Division of Surgery and Interventional Science Royal Free Hospital London UK; ^3^ Bioscience Education King's College London London UK; ^4^ Chelsea and Westminster Hospital London UK; ^5^ Great Ormond Street Hospital London UK; ^6^ Present address: Welsh Centre for Burns & Plastic Surgery Morriston Hospital Swansea UK

**Keywords:** human upper limb anatomy, muscle anomalies, radial dysplasia, soft tissue abnormalities

## Abstract

The defining features of the upper limb congenital anomaly, radial dysplasia (RD), involve both skeletal and soft tissue malformation, including muscle, vascular, and neural structures, with varying degrees of severity. Herein, we summarise the range of soft tissue anatomy malformations that have been described in RD, with the objective of better understanding their aetiology and clinical significance. Changes to muscle anatomy can include hypoplasia and fusion; vascular defects often involve persistent median arteries and absent radial arteries. There are also consistent differences in neural projections that have consequences on surgical approaches. Understanding these changes from normal anatomy, their origins and variations is important for improving diagnosis, management, and surgical outcomes in RD.

## Graphical abstract

We review the characteristic changes to the limb soft tissue and neurovascular abnormalities that have been described in the congenital limb birth defect, Radial Dysplasia. These include consistent changes in muscle anatomy or absence of specific muscles, persistent median arteries and absent radial arteries and consistent alterations in neural projections. We discuss the clinical significance in understanding these abnormalities, their origins and variations to improve diagnosis, management, and surgical outcomes in RD.

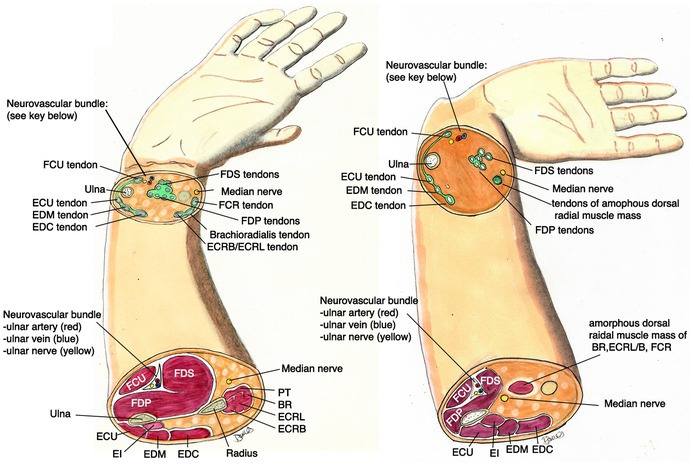

## 1 INTRODUCTION

Radial dysplasia (RD), also variously referred to as radial longitudinal deficiency (RLD), radial club hand (RCH) or radial ray deficiency/malformation, is an upper limb anomaly characterised by disordered development or abnormal organisation of the pre‐axial skeleton and associated soft tissues, ranging from hypoplasia (underdevelopment or reduced size due to incomplete formation) to aplasia (complete absence) of anatomical structures. Earliest published descriptions date to Petit JL (Petit, 1733). The entire length of the limb can be affected, but differences are most commonly apparent in the forearm and hand (Murphy et al., 2017; Takagi et al., 2017; van Nieuwenhoven et al., 2023). Deficiency of the radius extends from hypoplasia to complete aplasia and can be accompanied by varying degrees of ulna bowing and radial deviation of the hand/forearm angle. The instability at the wrist and malpositioning negatively affect limb function. Recurrence of radial deviation following surgery to stabilise the wrist and centralise hand position is a persistent problem, the causes of which are not understood (Ezaki, 2021; Murphy et al., 2017; Takagi et al., 2017; van Nieuwenhoven et al., 2023). Imbalance of the musculature and stiffer soft tissues could be responsible for the radial‐deviated, partially flexed hand position and bowed ulna that commonly presents in RD. Although rare in descriptions of RD, duplication anomalies, such as preaxial polydactyly and radial‐side duplications, can occur that fall within the radial ray deficiency spectrum (Goldfarb et al., 2020; Oberg et al., 2010). These are acknowledged here for completeness, but here we focus on soft‐tissue anatomy changes and deficiencies.

RD can be caused by exposure to teratogenic drugs or genetic mutations. It is associated with several genetic syndromes including Holt‐Oram, VACTERL, Fanconi anaemia, Townes‐Brocks and Thrombocytopaenia with Absent radius (TAR), however less than 30% of patients obtain a confirmed genetic diagnosis. The current literature focuses on and provides ample description and discussion of the characteristic skeletal malformation associated with RD. A thorough appraisal of the associated soft tissue defects, such as the morphology of the upper limb muscles, vasculature and innervation is comparatively scarce. Moreover, soft tissue and neurovascular abnormalities have a significant impact on functional impairment, clinical presentation, surgical management and treatment outcomes. Indeed, Wolff's law states that a bone will adapt to the stresses it is subjected to so that some of the bony deformities are secondary to the effect on the surrounding soft tissues. An understanding of the soft tissue and neurovascular changes characteristic of RD is important to enable clinicians to be aware of possible deviations from the normal anatomy that can influence limb biomechanics, and in turn, will influence clinical presentation, surgical management and possibly response to treatment, particularly recurrence of radial deviation (Ezaki, 2021; van Nieuwenhoven et al., 2023).

This narrative literature review is aimed at synthesising the anatomical soft‐tissue abnormalities associated with RD, including muscle, vascular, and neural anomalies, and summarising their clinical relevance. Since the anatomical literature on RD predates modern standards for systematic reviews, and because many early sources are descriptive rather than data‐driven, a narrative review was deemed most appropriate. A search of PubMed and Scopus identified publications describing soft‐tissue anatomy, embryology, and surgical findings in RD, RCH or RLD. Search terms included combinations of: ‘radial dysplasia’; ‘radial longitudinal deficiency’; ‘radial club hand’; ‘soft tissue malformation/abnormalities’; ‘muscle anomalies’; ‘nerve anomalies’; ‘vascular anomalies’. Searches were not limited by date to capture seminal early anatomical reports, including classical descriptions from the 18th–20th centuries. No language restrictions were applied, though non‐English texts were included only when English translations or secondary descriptions were available.

## 2 ABNORMALITIES IN MUSCLE MORPHOLOGY IN RADIAL DYSPLASIA

Detailed summary descriptions of the muscle anomalies seen in RD patients date back to the early 20th century, with the most recently published in 1969. Outlined below and summarised in Table 1, we have generated a contemporary compendium of the muscle anomalies seen in RD.

**TABLE 1:** Summary of muscle morphology in radial dysplasia.

Muscle	Schaeffer and Nachamofsky (1914) 1 specimen (2 limbs) dissected	Kato (1924) 3 specimens dissected report based on 250 cases	Forbes (1938) 1 specimen (1 limb) dissected	Heikel (1959) 7 specimens dissected report based on 64 cases including 2 TAR^a^ *hypoplasia*, *partial* or *total* aplasia refers to radial defect present	Pardini Jr. (1968) report based on 39 cases	Skerik and Flatt (1969) Based on previous dissection of 26 specimens No TAR patients included in series	Summary
Deltoid	Origin: normal Insertion: indirect—lateral intermuscular septum, continuous with teres major posteriorly; continuous with brachioradialis and ECRL/ECRB distally; and pectorals major anteriorly	Abnormally inserted with pectoralis or brachialis	Well developed, converges abruptly to be inserted into rough impression on lateral shaft of the humerus at junction of upper and middle third. The muscle is tendinous at its insertion and sends slip to lateral head of triceps	*Hypoplasia* ^a^: partially missing *Partial aplasia* ^a^: ventral part hypoplastic *Total aplasia* ^a^: normal	May be fused with triceps. Abnormal insertion	Normal in >2/3	Normal Tendinous slip to triceps, indirect insertions
Coracobrachialis	Origin: normal Insertion: abnormally extensive on humerus and brachial fascia	Mostly normal, may be fused with biceps proximally or abnormal insertion	Arises with short head of biceps as a single fused muscle mass from coracoid process	*Hypoplasia*: normal *Partial aplasia*: partly fused with biceps *Total aplasia*: fused with biceps; median innervation		Normal in >2/3. Frequently originates with short head of biceps	Normal but often fused with biceps median innervation
Biceps brachii	Origin: long head from joint capsule; short head normal Insertion: anterior and lateral aspect of distal humerus and coronoid process of ulna	Usually anomalous (absent, rudimentary or hypertrophied). Origin or insertion abnormal (lacertus fibrosus/bicipital aponeurosis only), fused with brachial or coracobrachialis	Short head arises with coracobrachialis Long head normal Short and long heads of biceps fused into a single muscle mass just below surgical neck of humerus, then divides into three bellies at the level of upper and middle third of humerus; lateral head passes around lateral shaft of humerus and fuses with lateral head of triceps. Forms triangular shape in close proximity to insertion of deltoid, likely a mass resulting from fusion of biceps and brachialis	*Hypoplasia*: origin (long head)—crest of greater tubercle; origin (short head): normal *Partial aplasia*: origin (short head): coracoacromial ligament and infraglenoid with tendon split; insertion distal‐medial humerus and radial tuberosity *Total aplasia*: long head absent in 4 of 5 cases; origin (short head)—coracoid process and infraglenoid tubercle	Most anomalous. Can be absent, rudimentary or hypertrophied. When radius is absent, it inserts into the lacertus fibrosus/bicipital aponeurosis	Long head almost always absent (if present, origin on anterior diaphysis of humerus); short head always present but rarely normal (fused with coracobrachialis at its origin or others more distally at its insertion in the forearm) and frequently has no terminal tendon and inserts into joint capsule and brachial fascia, muscles from either epicondyle or forearm flexors	Anomalous Often fused with coracobrachialis or brachialis, sometimes as a single mass Variable origin (glenoid or coracoid process) Variable insertion (elbow joint capsule, radial tuberosity, humerus epicondyles or forearm flexors) Median innervation
Brachialis	Absent but possibly incorporated with biceps brachii mass	Aplasia to hypoplasia. Fused with triceps. Abnormal insertion	Attached to lower half of anterior humerus and distally covers the bone from medial to lateral epicondyles; directly continuous with origins of FCR, FCU, PL, and EDC	*Hypoplasia*: normal; insertion: radial tuberosity *Partial aplasia*: hypoplastic; insertion in common with coracobrachialis *Total aplasia*: normal; origin—normal, insertion—lateral epicondyle + coronoid process; origin—normal, insertion—joint capsule; rudimentary; rudimentary		Can be normal, rudimentary, fused or absent but most frequently fused with biceps at its origin and no specific insertion site. Becomes continuous with common forearm flexors but can also insert into radial tuberosity, joint capsule or lateral epicondyle	Anomalous Origin: normal insertion: radial tuberosity, elbow joint capsule, lateral epicondyle, coronoid process
Triceps	Origin: long head—infraglenoid tubercle and significant portion of lateral border of scapula (fused with teres major); medial head—distal third dorsal humerus; lateral head—latissimus dorsi tendon Insertion: olecranon process of ulna	Often normal or fused with adjacent muscle	Normal except lateral head joined by slip from deltoid and one from coraco‐brachialis‐biceps mass	*Hypoplasia*: normal *Partial aplasia*: normal *Total aplasia*: normal in 5/5		Normal in >2/3. 1 case abnormal medial head	Normal can be fused with deltoid, brachialis or teres major
Brachioradialis	Origin: distal fibres of deltoid and overlying fascia Insertion: transverse carpal ligament and antebrachial fascia	Absent in almost 50%, fused with biceps or ECRL; insertion on ulna	Absent	*Hypoplasia*: origin—normal; insertion—common with ECRL; musculocutaneous innervation *Partial aplasia*: origin—normal; insertion—distal radial rudiment + intermuscular septa; median innervation *Total aplasia*: absent in 5/5	Absent in over 50%	Usually absent (reported by some) and only missing in total absence of the radius. 1 in 15 (Stoffel and Stempel) absent If present, inserts into radius or otherwise on the carpus	Absent Origin: normal Insertion: distal radius rudiment, intermuscular septa or carpus
Extensor carpi radialis longus (ECRL)	Origin: fused as an ECRL/ECRB mass from caudal portions of deltoid and triceps Insertion: transverse carpal ligament	Often absent; may be fused with EDC	Absent	*Hypoplasia*: origin—common with brachioradialis ECRB; insertion—normal; radial innervation *Partial aplasia*: origin is normal, insertion on distal radial rudiment + intermuscular septa *Total aplasia*: absent in 3/5; origin—coronoid process + fascia brachii, insertion—medial margin of carpus; origin—common with EDC, insertion base 4th MCP	Frequently absent. May be fused with EDC	Rudimentary or fused to adjacent extensors, but often missing	Frequently absent; if present can be fused with EDC/adjacent extensors Origin: lateral epicondyle/ supracondylar ridge Insertion: base 2nd MC, carpus, distal radial rudiment, 4th MC
Extensor carpi radialis brevis (ECRB)	Origin: fused as an ECRL/ECRB mass from caudal portions of deltoid and triceps Insertion: transverse carpal ligament	Often absent; may be fused with EDC	Origin: distal humerus down lateral edge of anterior ulna Insertion: flat tendinous expansion on the junction between middle and distal third of ulnar shaft	*Hypoplasia*: origin—common with brachioradialis ECRL; insertion—normal; radial innervation *Partial aplasia*: absent *Total aplasia*: absent; origin—lateral/distal half ulna, insertion 2nd + 3rd MCs; origin—mid‐ulna, insertion—dorsoradial margin of carpus, radial innervation	Frequently absent. May be fused with EDC.	The ECRB is absent in approximately 50% of cases. When present, it is typically fused with ECRL or shows anomalous insertion	Absent; fused with ECRL Origin: variable—lateral epicondyle, lateral edge proximal ulna, mid‐ or distal ulna
Anconeus	Origin: normal Insertion: whole of proximal half of dorsal surface of ulna	Normally present but often rudimentary	Normal			Absent in 50% or more	50% normal, others absent
Pronator teres (PT)	Absent	Often absent—rudimentary, fused with brachialis/biceps, insertion on ulna or radially on carpus	Deep in forearm because of continuity of biceps‐brachialis mass with PL and FCR. Insertion: lateral middle third of ulnar shaft (tendinous slip continues to lower end)	*Hypoplasia*: normal *Partial aplasia*: Rudimentary; origin—normal; insertion distally on radial rudiment; median innervation *Total aplasia*: origin—normal; insertion—intermuscular septa; median innervation; absent in 4/5	Frequently absent	Frequently fused with biceps‐brachialis mass, PL or FCR; usually inserts into rudiment of radius or into intermuscular septum	Anomalous Frequent fusion with biceps‐brachialis mass Insertion: radius rudiment, intermuscular septum or mid‐ulna
Flexor carpi radialis (FCR)	Origin: medial epicondyle of humerus (main) and fibres from biceps and intermuscular septum Insertion: ventral surface of distal ulna and carpus	Absent or rudimentary, fused with brachialis, biceps or other muscles of forearm	Origin: continuation of biceps‐brachialis mass on distal humerus Insertion: base of 2nd MC	*Hypoplasia*: origin—middle third radius; insertion—transverse carpal ligament and joint capsule *Partial aplasia*: origin—medial radial rudiment; insertion—normal *Total aplasia*: origin—proximal third ulna with FDS, insertion—radial side 2nd MC; origin—normal, insertion distal ulna and transverse carpal ligament; absent in 3/5	Frequently absent	Absent in approximately ≥50% of reported cases. When present, the structure shows marked anomalies (e.g., abnormal origin/insertion, hypoplasia, or fusion)	Frequently absent If present, fusion is common (brachialis, biceps or forearm flexors) Origin: can be distal humerus, radial rudiment, proximal/mid‐ulna Insertion: base 2nd MC, distal ulna or transverse carpal ligament
Palmaris longus (PL)	Origin: medial epicondyle of humerus but runs lateral to FCU Insertion: wrist joint capsule between ulna and carpus with FCU	Normal or absent, fused with FDS	Origin: medial to FCR arising as continuation of biceps‐brachialis mass Insertion: pisiform and hook of hamate	*Hypoplasia*: origin—normal, insertion—carpus and palmaris fascia *Partial aplasia*: origin—normal, insertion—wrist joint capsule *Total aplasia*: absent; origin—normal, insertion—pisiform; normal; absent		Absent in 50% or more. If present it is often fused with FDS/other flexors + aberrant insertion (more ulnar: pisiform, 5th MCP or soft tissue over 3rd/4th MCPs)	Absent Origin: normal/fused with biceps‐brachialis mass Insertion: carpus, joint capsule, palmaris fascia
Flexor carpi ulnaris (FCU)	Origin: normal Insertion: wrist joint capsule between ulna and carpus Insertion: distal anterior ulna and carpus	Usually normal. Insertion varies	Origin: medial epicondyle, medial side olecranon process and upper two‐thirds dorsal border of ulna Insertion: pisiform and expansion that fuses with ECU and dorsal carpal ligament	*Hypoplasia*: normal *Partial aplasia*: normal (hypoplastic) *Total aplasia*: normal in 5/5	Frequently present	Normal in >2/3. Origin normal but insertion occasionally aberrant	Normal Origin: normal Variable insertion
Flexor digitorum superficialis (FDS)	Origin: 2 humeroulnar heads from medial epicondyle of humerus (slight origin from intermuscular septum) and radial head from lateral border of ulna near carpus Insertion: heads joined in the hand, inserting on distal phalanx of index finger	Usually present but may be hypoplastic, fused with FDP, tendon to index finger often missing	Origin: anterior aspect of medial epicondyle + proximal ulna; insert on distal phalanges of middle + ring fingers	*Hypoplasia*: origin (humeral head‐HH) normal; origin (radial head‐RH)—distal radius; insertion—index finger *Partial aplasia*: origin (HH) normal; insertion—base middle/distal phalanx middle finger *Total aplasia*: origin (HH) normal; origin (RH) absent; origin (HH) normal, insertion—normal; RH absent; origin (HH) normal, insertion—middle/proximal phalanx of middle and ring fingers, RH missing; origin (HH) absent, insertion—base middle phalanx ring + little fingers; RH absent; origin (HH) normal	Frequently present	Present but often incomplete, atrophied or fused with flexor digitorum profundus. Tendon to index + little finger often absent. Radial head (origin) almost always absent	Present but incomplete (radial head absent), atrophied and fusion with FDP Origin: normal but radial head absent Insertion: missing tendons to index and little fingers and can attach to middle or distal phalanges
Flexor digitorum profundus (FDP)	Origin: whole anterior surface of ulna Insertion: distal phalanges of middle, ring and little fingers (tendons passed under FDS in the palm)	Varies less than FDS; tendon to index missing; insertion at origin of lumbricals	Origin: anterior + lateral aspects of proximal ulnar shaft Insertion: base of distal phalanx of middle, ring and little fingers (tendon to index finger is absent)	*Hypoplasia*: origin—normal; insertion—normal, index tendon missing *Partial aplasia*: origin—distal ulna, insertion—normal but index tendon absent *Total aplasia*: origin—normal; insertion—normal (middle finger tendon absent); origin—normal, insertion—base middle phalanx index, middle, ring finger but proximal in little finger	Frequently present	Tendon to index often absent. Insertions on base of proximal and middle phalanges	Present but fusion with FDS common Origin: normal but can occur at distal ulna Insertion: normal but often missing index tendon and can insert proximal phalanx
Flexor pollicis longus (FPL)	Absent; possibly part of undifferentiated mass	Majority absent but may be rudimentary or fused with deep flexors	Origin: anterior proximal ulna Insertion: radial aspect of base of second MC	*Hypoplasia*: absent *Partial aplasia*: absent *Total aplasia*: absent in 4/5; origin—distal ulna and volar carpus			Absent Origin‐ distal ulna Insertion: second MC
Pronator quadratus (PQ)	Probably mass of muscular tissue surrounding the distal ulna	Usually absent, may be rudimentary. Insertion on radial side of carpus or second MC	Origin: anterior aspect of lower half ulnar shaft Insertion: dorsum of radial side of carpus	*Hypoplasia*: normal; (hypoplastic, split into 2) Partial aplasia: absent *Total aplasia*: absent	Frequently absent	Absent in 50% or more. Usually totally absent but may be mass of muscle around distal ulna or may be found inserting into radial side of carpus or second MC	Absent Insertion: radial aspect of carpus
Extensor digitorum communis (EDC)	Origin: lateral epicondyle in common with EDM and from distal fibres of biceps brachii muscle and ante brachial fascia. Insertion: distal phalanges of index, middle, ring and little fingers	Often partly missing; fused with ECRL/ECRB or EDM	Origin: anterior aspect lateral epicondyle + biceps‐brachialis mass Insertion: divides into 2 tendons above wrist, under dorsal carpal ligament + into little and ring fingers	*Hypoplasia*: origin—normal, insertion—normal (tendon to index absent) *Partial aplasia*: origin—normal, insertion—tendon to index missing *Total aplasia*: origin—normal; insertion—ring + little fingers normal; origin—normal, insertion—base distal phalanx ring finger + base middle phalanx of little finger; origin—normal, insertion—base distal phalanx of index to little fingers; origin—normal, insertion—base middle phalanx of index to little fingers		Rarely absent but frequently fused with adjacent extensors (especially ECRL and EDM). Tendon to index frequently absent	Present but fused with adjacent extensors Origin: normal Insertion: base distal phalanx ring and little fingers (missing index and middle) Median > radial innervation
Extensor digiti minimi (EDM)	Origin: lateral epicondyle in common with EDC Insertion: tendon divided, giving one extension to EDC and the other became continuous with fascia overlying 5th MC	Not described	Not described	Not described	Not described	Normal in >2/3. Occasionally abnormal. If so, it is fused with either ECU or EDC distally	Normal but may be fused with ECU or EDC distally
Extensor indicis (EI)	Undifferentiated muscle mass	Absent or abnormal insertion	Origin: anterolateral aspect of distal ulna, under dorsal carpal ligament Insertion: extensor mechanism of index finger only	*Hypoplasia*: origin—dorsal mid‐ulna, insertion—normal *Partial aplasia*: origin—dorsal mid‐ulna; insertion—extensor apparatus middle and ring fingers *Total aplasia*: normal; origin—normal, insertion—extensor apparatus middle finger; origin—normal, insertion—base 3rd MCP; origin—normal, insertion—thin, diffuse tendons to index and carpus; origin—normal, insertion—base middle phalanx index finger and small tendons to carpus	Frequently absent	Present but abnormal insertion (middle and/or index fingers) onto any phalanx, metacarpal or carpal bones	Usually Present Origin: normal, mid‐ or distal ulna Insertion: variable to any phalanx of middle finger, MC or carpus
Extensor carpi ulnaris (ECU)	Origin: normal Insertion: broad fascial band on medial aspect of distal ulna	Usually normal	Origin: lateral epicondyle, dorsolateral ulna, and dorsal border radius, fuses with FCU distally and dorsal carpal ligament (perhaps this represents the ulno‐triquetral or ulno‐lunate ligaments) and a slip to base of 5th MC	*Partial aplasia*: origin—laterally on proximal ulna *Total aplasia*: origin—normal, insertion distal ulna; origin—common with EDC, insertion—2 tendons into distal ulna + base 5th MCP; origin—normal, insertion—2 tendons distal ulna; origin—normal, insertion—distal ulna and ulnar margin of carpus; origin—normal, insertion—diffusely on carpus		Normal in >2/3. Aberrant origin and insertion if abnormal (can be fused with EDC or FCU)	Normal Origin: normal Insertion: split into two tendons which can be to distal ulna, carpus or base 5th MC
Supinator	Absent	Frequently absent	Absent	*Hypoplasia*: normal *Partial aplasia*: origin—normal; insertion—intermuscular septa; median innervation? *Total aplasia*: absent in 4/5; origin—normal, insertion—intermuscular septa	Frequently absent	Not described	Absent Origin: normal if present Insertion: intermuscular septa, connective tissue between radial rudiment and carpus
Abductor pollicis longus (APL), extensor pollicis longus (EPL), extensor pollicis brevis (EPB)^a^	Probably within undifferentiated muscle mass	EPL absent	Absent but when present have abnormal insertions	Not described	EPL frequently absent	Not described (no TAR patients in this series)	
Abductor pollicis brevis (APB)^a^	Not described	Often missing/abnormal insertion	Not described	*Hypoplasia*: undefinable rudiment *Partial aplasia*: absent Total aplasia: absent; absent; origin—diffuse on carpus, insertion—diffuse laterally on index; origin—tendon APL/EPL, insertion—radially index; origin—tendon APL/EPB, insertion—radially index	Frequently absent	Not described	Often absent
Opponens pollicis (OP)	Not described	Absent	Not described	*Hypoplasia*: undefinable rudiment *Partial aplasia*: absent *Total aplasia*: absent	Not described	Not described	Absent
Flexor pollicis brevis (FPB)^a^	Not described	Absent	Not described	*Hypoplasia*: origin—normal but missing deep head; insertion—radial distal phalanx of index; median innervation. *Partial aplasia*: absent *Total aplasia*: absent in 2/5; origin—transverse carpal ligament, insertion—base of distal/middle phalanx of index/middle fingers; origin—carpus at base 3rd MCP, insertion—radially 3rd MCP; origin—pisiform bone, insertion—tendon EPB at base 2nd MCP, median innervation	Not described	Not described	
Hypothenar	Origin: undifferentiated triangular muscle sheet from transverse carpal ligament Insertion: medial border of 5th MC	Usually normal	Normal (small muscle from capitate to shaft of second MC)	*Normal*		Normal in >2/3. If abnormal can be present as a mass but undifferentiated	Normal
Interossei	Palmar: normal Dorsal: only one present to index finger	Usually normal/partially missing	Three dorsal (no dorsal interosseous to index finger), three palmar	Normal but in total aplasia there are only 3 dorsal, 2 volar	Frequently present	Normal in >2/3. If abnormal, most frequently first dorsal can be absent, rudimentary, atrophied or present but undifferentiated	Normal Dorsal interosseous to index finger absent
Lumbricals	Origin: undifferentiated muscle sheet given off FDS toward 5th MC Insertion: 2 muscles to ring and little fingers	Usually normal/partially missing	Not described	*Hypoplasia*: normal *Partial aplasia*: included in FDP *Total aplasia*: normal in 3/5; absent 1/5; origin—palmaris fascia, insertion—muscular to extensor expansion of middle/ring fingers only, tendinous to distal phalanx of ring finger	Frequently present	Normal in >2/3. If missing, most frequently to index	Normal

^a^ Frequently, the thumb is absent or non‐functional unless the patient has TAR syndrome and hence the limitation of data on these muscles.

In the shoulder girdle and arm of RD individuals, the deltoid, brachialis, and coracobrachialis muscles can be under‐ or over‐developed, entirely absent, abnormally inserted, or fused to adjacent musculature; but in the majority of cases, they are abnormal (Forbes, 1938; Heikel, 1959; Pardini Jr., 1968; Skerik & Flatt, 1969). When these anomalies are present, their severity appears independent of the degree of skeletal deficiency within the limb, suggesting that the embryological patterning of these muscles is, initially, a distinct process separate from skeletal development (Besse et al., 2020; Hasson et al., 2010). The triceps muscle is often normal, but it may be fused with neighbouring muscles, including deltoid or teres major; but fusion of triceps can also be seen with brachialis. The most frequently abnormal muscle in the arm is the biceps brachii: it can be absent, rudimentary, or hypertrophied. This muscle is often fused with coracobrachialis or brachialis as a single muscle mass with variable origins of the muscle heads (see Table 1). The tendon insertion of the biceps also varies and may insert at the elbow joint capsule, lacertus fibrosus (bicipital aponeurosis), radial tuberosity (its normal insertion), or distal radius if this is present (Forbes, 1938; Heikel, 1959; Schaeffer & Nachamofsky, 1914; Skerik & Flatt, 1969). In TAR syndrome specifically, the consistent presence of an abnormal brachialis that originates abnormally high on the humerus and inserts on the carpus, a so‐called ‘Brachiocarpalis’, has been described and is thought to contribute a deforming force for elbow and wrist (Oishi et al., 2009).

**FIGURE 1:**
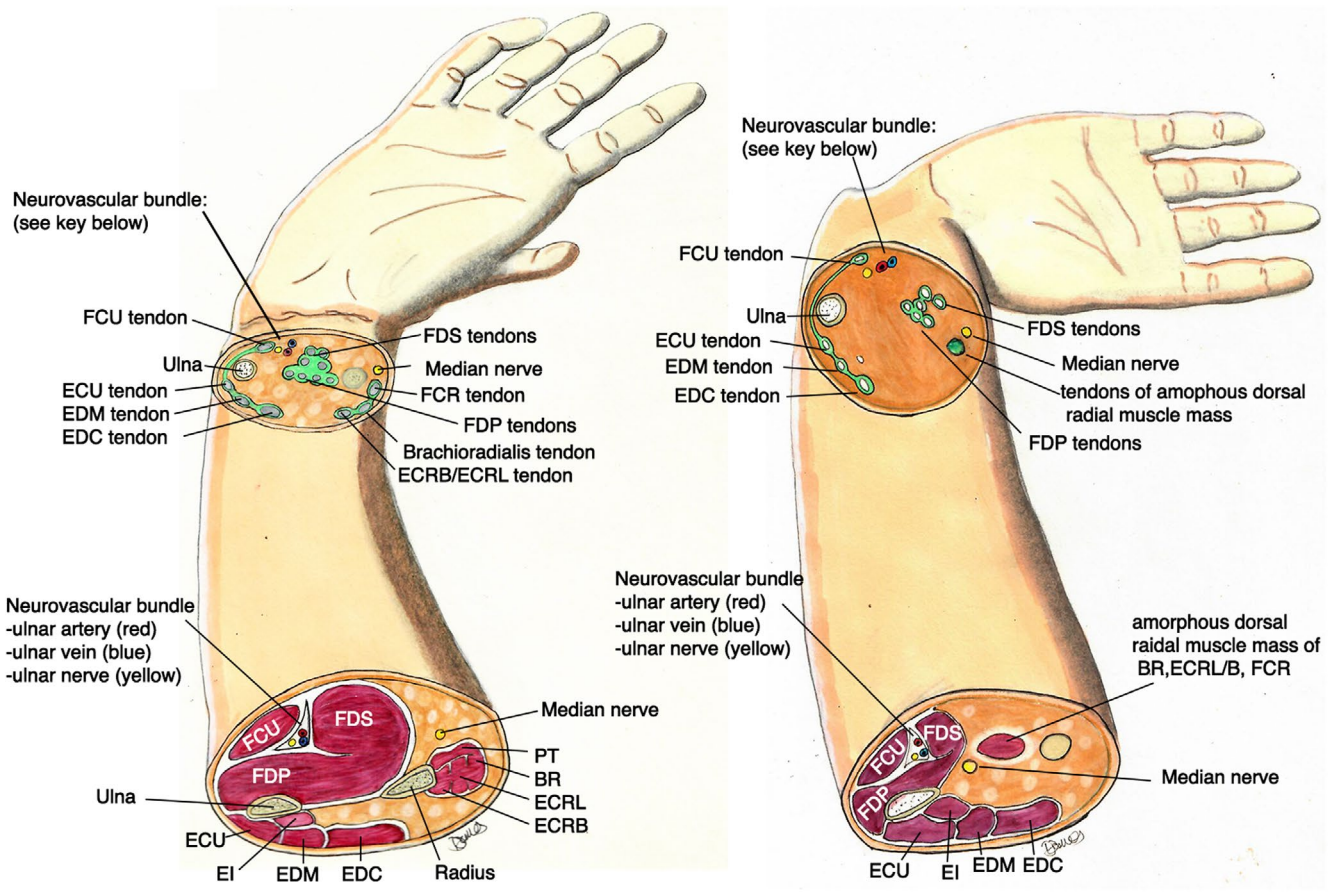
Graphical representation of some of the skeletal, muscular and neurovascular forearm anatomical changes commonly seen in RD: BR, brachioradialis; ECRL/ECRB, extensor carpi radialis longus/brevis; ECU, extensor carpi ulnaris; EDC, extensor digitorum communis; EDM, extensor digit minimi; FCU, flexor carpi ulnaris; FDP, flexor digitorum profundus; FDS, flexor digitorum superficialis; PT, pronator teres.

The forearms of RD patients have muscular abnormalities, as might be expected alongside the skeletal deficiency (illustrated graphically in Figure 1). Those constituting the dorso‐radial muscle mass contribute to the characteristic radial deviation and disequilibrium seen at the wrist. Brachioradialis, extensor carpi radialis longus (ECRL), extensor carpi radialis brevis (ECRB), supinator, extensor pollicis longus (EPL) and anconeus are usually missing or anomalous (fusion to other muscles, abnormal origins or insertions). Brachioradialis, ECRL and ECRB in normal individuals originate from the lateral supra‐epicondylar ridge and lateral epicondyle of the humerus and insert into the distal radius and second and third metacarpal respectively. Brachialis is a flexor of the elbow while ECRL and ECRB both extend and radially deviate the hand at the wrist joint. In RD, ECRL, and ECRB are usually present but are generally hypoplastic to varying degrees, fused together and may be abnormally inserted into the carpus or metacarpal bones. As a result, they compound the radial deviation, extensor lag and wrist instability (Forbes, 1938; Heikel, 1959; Lamb, 1977; Pardini Jr., 1968; Skerik & Flatt, 1969). The forearm flexors, pronator teres (PT), palmaris longus (PL) and flexor carpi radialis (FCR) are often absent but, when present, may be fused proximally with an aberrant biceps‐brachialis muscle mass (Forbes, 1938; Heikel, 1959; Pardini Jr., 1968; Schaeffer & Nachamofsky, 1914; Skerik & Flatt, 1969). This may be why, even in cases of Bayne type I RD, forearm rotation is reduced or absent. Where a thumb is present, it is hypoplastic to varying degrees and the normal attachment of the flexor pollicis longus (FPL), at the base of the distal phalanx of the thumb, is frequently absent (Smith et al., 2012).

Absence or hypoplasia of the thumb in RD is seen in over 80% of cases, with some studies putting this at 100% (Heikel, 1959; James et al., 1999; Lamb, 1977; O'Rahilly, 1951; Schaeffer & Nachamofsky, 1914). Currently, the most widely accepted phenotypic classification of the thumb hypoplasia is the Blauth classification, with notable modifications by Buck‐Gramcko, Manske and McCarrol (Table 2) and Smith (Blauth, 1967; Buck‐Gramcko, 2002; James et al., 1999; Smith et al., 2012).

**TABLE 2:** Modified Blauth classification of congenital thumb hypoplasia (Smith et al., 2012).

Grade	Features
I	Minor hypoplasia
II	Normal skeletal elements Narrow first webspace MCP joint instability Hypoplasia of thenar muscles
III	As per Type II as well as: Skeletal hypoplasia and aplasia of proximal metacarpal Radial carpal bones may be absent Aplasia of thenar and extrinsic muscles
IIIA	CMC joint present Narrow pencil‐like 1st metacarpal base, Rudimentary tendinous slips of extrinsic muscles, narrow 1st webspace, which may be partially syndactilised
IIIB	CMC joint absent As IIIA but base of 1st metacarpal is absent
IIIC	Absence of shaft and base of 1st metacarpal
IV	Absence of metacarpal with rudimentary phalanges Floating thumb
V	Total aplasia

Blauth Type I thumbs are structurally normal but smaller than those of unaffected individuals. Generally, these do not require surgical intervention as there is no significant functional problem. They may still have reduced strength and endurance on formal testing. Type II, in contrast, may require surgery as they display narrowing of the first webspace, deficiency of the intrinsic thenar muscles (this varies between hypoplasia and aplasia, particularly of the opponens pollicis) and instability of the metacarpophalangeal (MCP) joint, which all compound to adversely impact pinch function (Light & Gaffey, 2010). A further subclassification system for Blauth II deformities defines Type IIA by uni‐axial (ulnar) instability and type IIB by multiaxial instability of the MCP joint, which may help guide surgical management (Smith et al., 2012). The principal aims of surgery for these abnormalities must include reconstruction of the first webspace, stabilisation of the MCP joint and establishing adequate thumb opposition to ensure optimal hand function. Type III was subdivided into IIIA and IIIB by Manske, to consider carpometacarpal (CMC) joint stability. Type IIIA thumbs are characterised by the features present in type II as well as metacarpal hypoplasia and extrinsic tendon abnormalities (as outlined above, this is common in RD where any or all EPL, EPB, and APL muscles may be absent or abnormal) but with a CMC joint radiologically present. Type IIIB thumbs possess type IIIA features with the addition of an unstable CMC joint, but the shaft and head of the metacarpal are present. Buck‐Gramcko added an additional Type IIIC where only the head of the metacarpal remains. The relevance of these categories lies in the potential solutions for surgical reconstruction if it is attempted in these severely hypoplastic thumbs. Type IV thumbs, also known as floating thumbs or ‘pouce flottant’, have a neurovascular bundle, a nail and some distal bony support, but lack proximal bony support and have severely hypoplastic or absent soft tissue structures. Finally, type V refers to the complete absence of the thumb (Light & Gaffey, 2010). This classification splits what is a spectrum of abnormality into groups that help in deciding surgical management.

The intrinsic hypothenar muscles of the hand are usually present in RD and exhibit a normal morphology whilst the contralateral thenar muscles are usually missing, as may be expected given the high frequency of thumb hypoplasia or absence (Forbes, 1938; Heikel, 1959; Pardini Jr., 1968; Schaeffer & Nachamofsky, 1914; Skerik & Flatt, 1969). However, one study reported that flexor pollicis brevis (FPB) was absent in only 12 out of 22 cases, abnormal in 8 and normal in 2 (Skerik & Flatt, 1969), which may be related to its innervation, which can be from the ulnar nerve. Furthermore, while FPB origin can be normal it may be missing the deep head, arise from the transverse carpal ligament or from the carpus itself (Heikel, 1959). Similarly, in two studies, the adductor pollicis (AdP) was absent in 23 out of 29 cases and opponens pollicis (OP) was absent in 22 out of 29 cases (Heikel, 1959; Skerik & Flatt, 1969). The interossei and lumbricals are usually normal but can be incomplete. Consistent with the radial bias, the most frequently absent muscles are those that normally insert onto the radial aspect of the index finger, that is, first dorsal interosseous and first lumbrical. This has significant implications for the strength of abduction and, therefore, of opposition, in index finger pollicisation (Heikel, 1959; Pardini Jr., 1968; Skerik & Flatt, 1969). Interestingly, the anatomical literature does not include the presence of ‘pollex abductus’, a congenital malposition of the FPL tendon that can occur in thumb hypoplasia and is important to the surgeon. The anomalous tendon travels radial to the metacarpophalangeal (MP) joint and bifurcates at mid‐phalangeal level with a major insertion passing obliquely to the volar base of the distal phalanx whilst a lesser insertion passes dorsally into the EPL tendon. This results in the elimination of normal active extension at the interphalangeal (IP) joint so that contraction of FPL leads to abduction forces on MP and IP joints, made worse by the laxity of the ulnar collateral ligament (Lister, 1991).

A normal thumb is one with normal skeletal architecture, intrinsic, and extrinsic muscle function, joint stability, and opposition mechanics. In a clinical situation, the presence of a normal thumb in the presence of an apparent RD should cause the clinician to question the diagnosis. However, a review of 250 patient cases between 1733 and 1923 documents 159 hypoplastic or absent thumbs in 356 affected limbs (44%) of patients with RD (Kato, 1924). Similarly, in a cohort of 62 Holt‐Oram syndrome patients (114 affected limbs), there were 79 hypoplastic or absent thumbs (69%) but 27% of this cohort was not classifiable by Blauth classification (Wall et al., 2015). It may be that since this is a spectrum disorder, the literature, particularly older publications, has included thumb hypoplasia with RD thereby not documenting accurately the thumb hypoplasia, and by inference, suggesting RD patients can have normal thumbs. By definition, RD patients do not have normal or functional thumbs, apart from those with TAR syndrome, who have functional but abnormal thumbs (Goldfarb et al., 2007). That said, some of the changes in the thumb may be subtle, such as a narrow 1st web disguised by ulnar collateral laxity of the MP joint or minor hypoplasia of APB and may be easily overlooked without detailed examination.

In addition to the thumb, other radial anatomy, in particular, the index and middle fingers, is also commonly affected. For example, the flexor digitorum superficialis (FDS) and flexor digitorum profundus (FDP) tendons, although present within the radial digits, are often abnormal to varying degrees. FDS is sometimes incomplete with an absent radial slip or entirely fused with the FDP (Heikel, 1959; Skerik & Flatt, 1969). When present within the index finger, the FDP tendon is often anomalously inserted into the base of the middle or proximal phalanx (Heikel, 1959; Skerik & Flatt, 1969). Furthermore, a number of anatomical studies (with a combined total of 75 examined limbs) have demonstrated complete absence of both tendons within the index finger (Forbes, 1938; Heikel, 1959; Pardini Jr., 1968; Schaeffer & Nachamofsky, 1914; Skerik & Flatt, 1969). This would explain clinical cases where the index finger is found to be stiff, with an absence of interphalangeal joint creases with flexion only at the MCP joint.

In the distal part of the forearm, pronator quadratus (PQ) is absent in over half of RD cases but when present, may exist as a muscle mass around the distal ulna or insert into the lateral aspect of the carpus (Forbes, 1938; Skerik & Flatt, 1969). In the posterior compartment, the supinator is frequently absent (consistent, together with a potentially abnormal biceps, with the usual pronated position of the forearm) whilst the extensor carpi ulnaris (ECU) is usually normal (Forbes, 1938; Heikel, 1959; Skerik & Flatt, 1969). Hence, in the past, ECU has been utilised as a recipient site for tendon transfers in corrective wrist stabilisation procedures for RD. Here, it can act to reduce the risk of recurrent radial deviation but is unlikely to alter the flexion aspect of the deformity. Extensor digitorum communis (EDC) is present but can be fused to adjacent extensors. Its origin tends to be unaffected but its insertion may vary into the proximal and middle phalanges (Skerik & Flatt, 1969). Extensor indicis (EI) can be absent but, when present, its insertion is likely to be anomalous and attach to any of the index phalanges, metacarpal or carpus. It is often seen as hypoplastic and its suitability for tendon transfer is limited. The EPL, extensor pollicis brevis (EPB) and abductor pollicis longus (APL) muscles are seldom described in the literature but when they are, they are often absent with four studies showing absence of each of these muscles in 13 out of 22, 18 out of 22 and 14 out of 22, respectively (Forbes, 1938; Heikel, 1959; Pardini Jr., 1968; Skerik & Flatt, 1969).

## 3 ABNORMALITIES IN ARTERIAL MORPHOLOGY IN RADIAL DYSPLASIA

The documented abnormalities of the main arteries of the upper limb are summarised in Table 3.

**TABLE 3:** Summary of arterial morphology in radial dysplasia.

Vessel	Schaeffer and Nachamofsky (1914)	Kato (1924)	Forbes (1938)	Pardini Jr. (1968)	Skerik and Flatt (1969)	Hadidi et al. (1990)	Inoue and Miura (1991)
Brachial	Emerges medially at canal formed by fibres of biceps brachii that lay over an area between the epicondyles of the humerus	Usually normal but may divide into two branches in the arm. Profunda brachii may come off posterior circumflex artery	Does not divide into radial and ulnar, but rather continues as ulnar artery	Usually normal	Usually normal but may divide into two high in the arm or not at all at the elbow. Deep brachial may come off posterior humeral circumflex	Not described	Not described
Ulnar	Not described	Usually present but may be anomalous	Between two heads of PT. Gives off large branch which passes down between FDP and PT (probably corresponds to anterior interosseous)	Usually normal	Usually present + normal. May be anomalous, concomitant with absence of radial artery	Not described	Present in all cases with 1/13 unusual division at distal third of forearm
Radial	Not described	Very frequently absent or small	Not described	Frequently absent or very small calibre	Frequently absent. If present, usually small and associated with displacement of ulna	Not described	Absent in 6/13, 2 normal, 5 small. High division into 2 branches proximal to intercondylar line of humerus in 2/13
Interosseus	Not described	Well developed and may replace radial/ulnar arteries	Not described	Not described	Not described	Not described	Not described
Median	Not described	Not described	Not described	Not described	Not described	Not described	Present in 10/13
Superficial palmar arch	Not described	Not described	Not described	Not described	Not described	Atypical superficial palmar arch in all cases	Absent in 2/13
Deep palmar arch	Not described	Not described	Not described	Not described	Not described	Not described	Small or absent in 12/13

The brachial artery is usually present and normal (Pardini Jr., 1968). However, it may divide into two vessels within the proximal arm, whilst the profunda brachii, usually a branch of the brachial artery, can arise from the posterior humeral circumflex artery. Similarly, the ulnar artery is usually present and normal but can be anomalous (Skerik & Flatt, 1969). One study described this vessel arising between the two heads of PT, which then gave off a large branch that passed down between FDP and PT, possibly representing the anterior interosseous artery (Forbes, 1938). Indeed, the interosseous artery is often well developed in RD and interestingly, may replace the radial or ulnar arteries, or both (Pardini Jr., 1968; Skerik & Flatt, 1969). Furthermore, an arteriographic study of 13 cases with radial deficiencies noted the presence of an unusual division of the ulnar artery, in the distal third of the forearm into two branches distinct from its normal division in the proximal forearm (giving rise to the anterior interosseous artery) and at the level of the carpus that go on to supply the superficial and deep palmar arches (Inoue & Miura, 1991). More distally, the superficial palmar arch was absent in 2 out of 13 cases, whilst the deep palmar arch was small or absent in 12 out of 13 cases of RD (Inoue & Miura, 1991). In contrast, the radial artery is frequently absent or has a small calibre (Inoue & Miura, 1991; Pardini Jr., 1968; Skerik & Flatt, 1969). This might be unsurprising given the radial bias to the severity of characteristic RD anomalies. However, one study found that in all cases of ulnar ray deficiency studied (*n* = 4), the radial artery was smaller whilst the ulnar artery was dominant, when the reverse might be expected (Hadidi et al., 1990). Strikingly, the median artery (a transient embryonic axis artery of the upper limb) persisted in 10 of 13 cases in line with an incidence of 53.3% in radial deficiency (Inoue & Miura, 1991). In contrast, the prevalence of a persistent median artery in the general population ranges from 1.1 to 16%, which normally regresses in the second month of embryonic life to become a thin vessel known as commitans nervi median or palmaris profundus (Singla et al., 2012). This is relevant when considering free tissue transfer in surgical reconstruction, as the options for anastomosis may be to the side of the main supply to the hand or to anastomose to the median artery.

## 4 ABNORMALITIES IN NERVE SUPPLY OF THE UPPER LIMB IN RADIAL DYSPLASIA

An early study describes the brachial plexus as largely normal in RD patients in terms of distribution but that its main trunks are unusually large compared to normal individuals (Schaeffer & Nachamofsky, 1914). Given the muscle hypoplasia or aplasia seen in RD, nerve trunks might be expected to be smaller. A later study has confirmed these findings, however, and adds that some of the terminal branches receive contributing fibres from higher cervical segments (Skerik & Flatt, 1969). The axillary nerve is rarely mentioned in the literature but it has been described as normal (Skerik & Flatt, 1969). While some studies have reported the musculocutaneous nerve terminating within the biceps muscle in RD (Heikel, 1959; Schaeffer & Nachamofsky, 1914), others have reported its complete absence (Forbes, 1938; Pardini Jr., 1968). These findings are corroborated by other studies that describe the musculocutaneous nerve as the most commonly absent nerve in RD and that when present, it is usually abnormal; either joined with or substituted by the median nerve (Kato, 1924; Skerik & Flatt, 1969). In the absence of the musculocutaneous nerve, the median nerve supplies the anterior compartment of the arm and tends to substitute the innervation of muscles usually supplied by musculocutaneous, radial or ulnar nerves in the forearm, whenever these are missing (Forbes, 1938; Pardini Jr., 1968; Schaeffer & Nachamofsky, 1914; Skerik & Flatt, 1969). Interestingly, one study found that the median nerve had components from both the medial and posterior cords, which remained independent down to the level of the curvature of the elbow, then uniting to become the median nerve proper (Schaeffer & Nachamofsky, 1914). Moreover, other studies describe the median nerve as having a wide range of variations in its course as well as anastomoses with musculocutaneous nerve, innervation of extensors of the forearm or muscles in the anterior compartment of the arm (see Table 4) (Skerik & Flatt, 1969). One study describes eight variations of the median nerve (Stoffel & Stempel, 1909):

- Very superficial course along the radial edge of the brachioradialis muscle until reaching and entering the palm.
- Deep to PL and FDS muscles to the transverse carpal ligament.
- Along the lateral edge of FDS to the hand.
- At the elbow, it travelled deep to the flexors and supplied them before reappearing superficially mid‐forearm between brachioradialis and EDC.
- After significant contributions to flexors in the arm, it coursed underneath PT and deep to flexors, supplying the area. It then surfaced at the volar aspect of the wrist where it gave off a dorsal branch to the hand.
- Supplied arm flexors and then surfaced between FDS and brachioradialis, going further to supply the radial aspect of the palm.
- After sending branches to the arm flexors, it travelled through and supplied brachioradialis. In the forearm, it followed a normal course except for piercing PT and running between FDS and FDP.
- Supplied arm flexors and a sensory branch to supply the area normally innervated by the antebrachial cutaneous nerve by piercing through the brachioradialis muscle.

**TABLE 4:** Summary of nerve morphology in radial dysplasia.

Nerve	Schaeffer and Nachamofsky (1914)	Kato (1924)	Forbes (1938)	Heikel (1959)	Pardini Jr., 1968	Skerik and Flatt (1969)
Musculocutaneous	Ended in substance of biceps muscle (also small nerve from lateral cord into coracobrachialis)	Often missing, substituted by median nerve	Absent	Hypoplasia: substitutes superficial radial nerve, otherwise normal Partial aplasia: Total aplasia: normal motor innervation, joins median nerve	Usually absent	Most frequently missing nerve. If present, usually anomalous, either joined with or substituted by the median. Joined median nerve in 2 cases (Heikel). Hypoplasia 5/7 present; partial aplasia 3/3 present; total aplasia 3/14 present
Axillary	Not described	Not described	Not described	Not described	Not described	Rarely mentioned; described as normal
Median	Components from both medial and posterior cords	Takes over function of radial or ulnar nerve when these are missing	Between 2 heads of PT, deep surface of FDS then radially Supplies anterior compartment of the arm in absence of musculocutaneous nerve Sensory innervation to index, middle and radial side of ring finger	Hypoplasia: normal (also innervating skin of thumb) Partial aplasia: substitutes musculocutaneous. Anastomoses at elbow with superficial radial nerve, supplies radial‐volar border of thumb to ring fingers, ulnar border of ring finger and dorsal little finger Total aplasia: anastomoses with musculocutaneous distally on upper arm, innervates extensors of forearm, otherwise normal	One of most superficial structures on radial aspect of forearm. Supplies anterior compartment of arm in absence of musculocutaneous and will substitute for terminal distribution of the radial, providing both motor and sensory innervation to these areas.	Many variations, depending on status of the other nerves Supplies anterior compartment of the arm (in absence of musculocutaneous) and usually substitutes for terminal distribution of radial nerve. Riordan: frequently most superficial structures on radial aspect of forearm. Stoffel & Stempel (16 cases) described eight variations in its route. Median provides sensation to radial side of hand and anastomoses with sensory branch of ulnar on the dorsum
Radial	Present but no description.	Missing or ends at elbow joint (substituted by median or ulnar)	Ends abruptly just above lateral epicondyle	Hypoplasia: superficial branch missing, otherwise normal Partial aplasia: superficial branch fuses into median nerve, otherwise normal Total aplasia: ends at elbow joint, substituted by median nerve	Usually ends at the elbow after supplying the triceps	Most describe it ending just above the lateral epicondyle after innervating triceps Does not specify which muscles supplied by deep branch of radial nerve. If superficial present, it sometimes pierces brachioradialis or entangled in it. Superficial radial nerve presence in Hypoplasia 5/6; partial aplasia 1/2; total aplasia 0/9
Ulnar	Distal half of ulna, runs between FCR (lateral) and PL (medial)	Not described	Normal	Not described	No description but normally present and innervates muscles normally supplied by the radial	Usually present + normal May innervate entire FDP + sensory distribution may be greater

Furthermore, the median nerve is often the most radial structure seen at the wrist level and so is vulnerable to injury during corrective wrist stabilisation surgery. For this reason, it is sometimes referred to as the ‘radian nerve’, reflecting its consistently anomalous radially situated position. Its site makes it frequently the tightest structure on the radial side of the wrist and the main limiting factor in correction of the wrist position in RD. These variations in anatomical positioning and branching of the median nerve and its tightness are important to be aware of when approaching surgery in RD, particularly in recurrent cases where the nerve may be encased in scar tissue.

The radial nerve has been described as suddenly terminating at the level of the elbow joint after innervating the triceps muscle (Kato, 1924). However, it can be normal in some cases, and in others, wholly or partially absent (Forbes, 1938; Heikel, 1959; Pardini Jr., 1968; Skerik & Flatt, 1969). In contrast, the ulnar nerve is usually present and normal (Forbes, 1938; Heikel, 1959; Pardini Jr., 1968; Skerik & Flatt, 1969). However, in the absence of the radial or median nerve it may innervate the muscles usually supplied by these two separate nerves. Furthermore, it may be observed more superficially at the level of the distal half of the ulna running between FCR (laterally) and PL (medially) as well as slight variations of motor and sensory innervation, which are summarised in Table 4 (Forbes, 1938; Heikel, 1959; Pardini Jr., 1968; Skerik & Flatt, 1969).

## 5 DISCUSSION

In the absence of a part or whole of a bone, such as occurs in RD, it is reasonable to predict that the morphology of the muscle could be affected. It is clear, however, that in RD there are abnormalities in muscles at sites where there is no associated skeletal abnormality. This fits with the clinical knowledge that the whole of the upper limb may be affected although frequently little difference in skeletal structures is seen more proximally. Despite the abnormalities described for the shoulder and elbow, instability in the shoulder is relatively uncommon in RD patients and inability to flex the elbow is even rarer. Indeed, patients often rely on elbow flexion for much of their function, and those with TAR syndrome frequently have a flexion deformity at the elbow. Review of the reported soft tissue defects in RD reveals some consistent features that together with most recent data from experiments on animal models suggested that they cannot simply be explained by the absence of muscle insertion sites (Besse et al., 2020; Hasson et al., 2010). In the absence of an insertion site during embryonic stage, developing muscles can make consistent and predictable decisions on where to insert at alternative sites. For example, in RD, the biceps brachii muscle often does not form a terminal tendon and inserts into the elbow joint capsule and antebrachial fascia, whilst ECRB and ECRL, when present, insert at or near the carpus (distal ulna, base of metacarpal bones or transverse carpal ligament) (Forbes, 1938; Heikel, 1959; Schaeffer & Nachamofsky, 1914; Skerik & Flatt, 1969). These findings suggest that in the absence of their normal insertion sites, muscles can respond to other guidance cues and follow paths that do not appear to be random. This suggests the presence of multiple guidance cues in the limb environment and raises fundamental questions: How do nascent muscles ‘know’ where to attach consistently to the same place in normal individuals? Does this process rely on a particular target or signal? Are these cues intrinsic to the muscle itself, the target or a combination of both?

In addition, little is known about the mechanisms that control the splitting of individual bundles into smaller units, such as is observed in the formation of ECRL and ECRB. Strikingly, this event frequently fails to occur in RD so that, when present, these muscles are fused as a single muscle mass. In the mouse model, conditional deletion of *Tbx5* in the muscle connective tissue (MCT) results in the disruption of cellular events that lead to a spectrum of soft tissue patterning defects ranging from complete absence, muscle dysplasia and failure of muscle splitting (Besse et al., 2020; Hasson et al., 2010). The splitting process in the mouse normally occurs from distal to proximal, forming two ‘daughter’ muscles from a single ‘parental’ bundle and this event does not occur after conditional deletion of *Tbx5* in the MCT (Besse et al., 2020). Strikingly, the equivalent splitting process occurs in human embryonic muscle formation (Wilde et al., 2021) and failure of ECRL and ECRB to split is a feature observed in HOS and other presentations of RD (Table 1 above). Muscles, tendons and fascia are frequently disorganised and poorly formed in RD (Murphy et al., 2024), and these defects appear to arise from an inherent defect in the connective tissue fibroblasts that surround the muscle fibre precursors and ultimately form the endo, peri and epimysium myofascial layers that provide structural integrity to muscle bundles (Besse et al., 2020; Hasson et al., 2010; Kardon et al., 2003; Mathew et al., 2011; Murphy et al., 2017). Extended to humans, this has implications for surgical management as disorganised tissue may have different material properties and may not act well as a tendon transfer.

Unlike other muscle groups, FDS in the mouse is initially found in the forelimb autopod and later elongates and translocates proximally to become an extrinsic muscle in the zeugopod of the forelimb (Huang et al., 2013). We have shown recently that a similar translocation occurs in humans (Wilde et al., 2021), but this appears to be disrupted in RD resulting in the muscle being found incomplete or fused to FDP, perhaps owing to the absence or incomplete cues in the MCT and/or failure of FDS to insert proximally. The abnormalities of the tendons in the radial side of the forearm are important to be aware of when performing a release of the wrist prior to any form of stabilisation. The absence of supinator accounts for the limited, if any, forearm rotation present and makes attempts to recreate forearm rotation in those with a partial radius likely to be doubtful of success. Similarly, the absence or hypoplasia of the first dorsal interosseous muscle has implications for the results of index finger pollicisation as it is ideally utilised in the creation of an abductor for the new thumb. Not all cases require pollicisation, but in cases where pollicisation is indicated, absence of a good abductor increases the likelihood of needing a secondary opponensplasty and its absence questions whether this should be planned for primarily. The absence of EIP is well recognised and methods of pollicisation have accounted for this, which is why it is rarely used in thumb hypoplasia as an opponensplasty. The combined defects of muscle and fascia found in RD may contribute deforming forces that lead to ulna bowing and the frequent but variable recurrence of radial deviation of the hand that frequently occurs after surgery to stabilise the wrist and centralise hand position (Ezaki, 2021; Takagi et al., 2017; van Nieuwenhoven et al., 2023).

Vascular supply to the upper limb first arises from a capillary network originating in the dorsal aorta, which expands into the developing limb bud prior to the nerves. The initial network of capillaries is replaced by axial arteries that develop from proximal to distal through a combination of maintenance and remodelling of some vessels and regression of others (Rodriguez‐Niedenfuhr et al., 2001). One such transient axial vessel, the median artery, persists at a higher incidence in RD compared with normal individuals, which may be, at least in part, due to the lack of the appropriate cues that also guide muscle development (Inoue & Miura, 1991). The radial artery is frequently absent, and the persistence of the median artery is important when considering placement of pins for soft tissue or bony distraction and for consideration of free tissue transfer as a suitable recipient vessel needs to be identified. The abnormal radial supply may also be relevant to the tendency in Holt‐Oram patients for early epiphyseal closure in the proximal phalanx of the index finger after pollicisation, producing an abnormally short thumb (Lochner et al., 2012).

At Carnegie stage (CS) 15, nerves start to enter the limb bud and become recognisable as far as the hand by CS17 (Rodriguez‐Niedenfuhr et al., 2001). Like the radial artery, the radial nerve is frequently absent and replaced by the median nerve, which will innervate muscles normally supplied by the radial nerve (Forbes, 1938; Heikel, 1959; Kato, 1924; Pardini Jr., 1968; Skerik & Flatt, 1969). This suggests an underlying link in the development or patterning of these soft tissues within the upper limb but also that, in the absence of such cues, there is a mechanism by which structures, such as the median nerve, can still innervate developing muscles within the upper limb. The radially sited position of the median nerve makes procedures such as fixator application and soft tissue release in these patients at risk of significant and devastating nerve injury. This should be remembered when exploring these cases, especially secondarily when the nerve may be encased in scar tissue and less likely to be easily recognised and, therefore, more likely to be inadvertently injured. Abnormal branching of the median nerve distally may make standard approaches to the distal radius in those patients with lesser degrees of hypoplasia more hazardous. Even carpal tunnel decompression may be less straightforward due to this and the potential for a persistent median artery which will confuse most surgeons, making them question whether they have inadvertently entered Guyon's canal.

## 6 CONCLUSION

Data from animal models are beginning to shed light on the developmental mechanisms that control the formation of individual muscle bundles, their insertion to the limb skeleton via specific tendons and neurovascular supply. All the precursors of limb muscles originate from somites, blocks of tissue on either side of the developing spinal cord and must migrate into the developing limb bud, where they receive instructive cues that dictate their final position and the muscle bundle they will contribute to. A critical source of such signals are the connective tissue fibroblasts that surround the muscle fibre precursors and ultimately form the endo, peri, and epimysium myofascial layers. Similarly, neurovascular development and patterning also appear to rely on morphogenetic cues and could be the same as those affecting muscle development. It is these intrinsic signals that appear to be disrupted in RD and contribute to its complex clinical presentation. The resulting abnormal anatomy of soft tissue could subsequently contribute to limitations in range of motion and recurrence of radial deviation.

## Data Availability

Data sharing not applicable to this article as no datasets were generated or analysed during the current study.
